# Dose-dependent vitamin D3 supplementation attenuates endothelial dysfunction and renal injury in an obstructive sleep apnea rat model

**DOI:** 10.3389/fphar.2026.1847011

**Published:** 2026-09-15

**Authors:** Abdulmueen A. Alotaibi, Syed Shahid Habib, Hayam Gad, Tamadar Al Doheyan, Mubarak Aldosary, Alanoud T. Aljasham, Asma F. Alotaibi, Yasser A. Alshawakir, Fuzail Ahmad, Abeer A. Al-Masri

**Affiliations:** 1 Department of Physiology, College of Medicine, King Saud University, Riyadh, Saudi Arabia; 2 Anesthesia Technology Department, Almaarefa University College of Applied Sciences, Riyadh, Saudi Arabia; 3 Department of Pathology, College of Medicine, King Khalid University Hospital, King Saud University, Riyadh, Saudi Arabia; 4 Department of Pharmaceutical Care, Ministry of National Guard Health Affairs, Riyadh, Saudi Arabia; 5 Department of Clinical Laboratory Sciences, College of Applied Medical Sciences, King Saud University, Riyadh, Saudi Arabia; 6 Department of Pharmacology and Toxicology, College of Pharmacy, King Saud University, Riyadh, Saudi Arabia; 7 Experimental Surgery and Animal Laboratory, Prince Naif bin Abdulaziz Health Research Center (PNHRC), College of Medicine, King Saud University, Riyadh, Saudi Arabia; 8 Respiratory Therapy Department, Almaarefa University College of Applied Sciences, Riyadh, Saudi Arabia

**Keywords:** cholecalciferol, endothelial dysfunction, endothelin-1, E-selectin, ICAM-1, intermittent hypoxia, nitric oxide, obstructive sleep apnea

## Abstract

**Background:**

Endothelial dysfunction driven by intermittent hypoxia (IH)-induced oxidative stress, nitric oxide depletion, and adhesion molecule upregulation underlies obstructive sleep apnea (OSA)-related cardiovascular complications. Vitamin D deficiency, which frequently coexists with OSA, may further amplify this endothelial injury through impaired endothelial nitric oxide synthase (eNOS) regulation and heightened renin-angiotensin-aldosterone system (RAAS) activity; however, the dose-dependent therapeutic potential of vitamin D3 on combined endothelial and renal outcomes in an IH model remains uncharacterized.

**Methods:**

Fifty-six adult male Sprague-Dawley rats were allocated to seven groups: control, vitamin D-deficient (VDD), VDD with severe IH (SIH-VDD), and four supplementation subgroups receiving high-dose (37.5 μg/kg/day) or low-dose (25 μg/kg/day) cholecalciferol under severe or moderate IH for 14 days. Serum E-selectin, ICAM-1, ET-1, NO, and PCSK9 were quantified by ELISA. Renal histopathology was assessed on H&E sections by a blinded pathologist. Data were analyzed by one-way MANOVA, univariate ANOVA, and LSD *post hoc* tests (p < 0.05).

**Results:**

The SIH-VDD group showed the most severe endothelial activation: peak E-selectin (4.92 ± 0.90 ng/mL), ICAM-1 (201.50 ± 21.55 ng/mL), ET-1 (5.31 ± 0.81 pg/mL), and PCSK9 (213.50 ± 14.71 ng/mL), with the lowest NO (14.88 ± 2.16 μmol/L; all p < 0.001 vs. control). High-dose supplementation produced substantial recovery across all markers; PCSK9 normalized to near-control (162.50 ng/mL) under severe IH. Low-dose supplementation yielded incomplete normalization. Renal histopathology revealed mild interstitial lymphocytic infiltration in IH-exposed groups, with proximal tubular vacuolisation exclusively in the SIH-VDD group and entirely absent in all supplemented groups. Glomerular architecture remained preserved throughout.

**Conclusion:**

Vitamin D deficiency combined with IH upregulates endothelial dysfunction markers and induces renal tubular injury in an experimental OSA model. High-dose vitamin D_3_ attenuates these changes dose- and severity-dependently, supporting its role as a pharmacological adjunct for OSA-associated endothelial and renal complications.

## 1 Introduction

Obstructive sleep apnea (OSA) is a chronic sleep disorder characterized by repetitive episodes of complete or partial upper airway collapse during sleep, culminating in cycles of intermittent hypoxia (IH), hypercapnia, and arousal (Jang et al., 2024). Beyond its neurological sequelae, OSA exerts profound cardiovascular (CV) effects through heightened sympathetic activity, systemic inflammation, oxidative stress, and endothelial injury (Lavie, 2015). Epidemiological data consistently identify OSA as an independent risk factor for hypertension, coronary artery disease, heart failure, and stroke (Iannella et al., 2025). The pathological vascular hallmark of OSA is endothelial dysfunction arising from IH-driven oxidative stress, cytokine release, and vasoactive mediator dysregulation (Harańczyk et al., 2022).

The endothelium maintains vascular homeostasis principally through constitutive nitric oxide (NO) production by endothelial nitric oxide synthase (eNOS), which inhibits platelet aggregation, leukocyte adhesion and promotes vasodilation (Tran et al., 2022). In OSA, recurrent oxidative burden leads to eNOS uncoupling, reactive oxygen species (ROS)-mediated NO scavenging, and a pro-inflammatory, pro-thrombotic vascular state (Lavie, 2015). Among established circulating markers of endothelial activation, intercellular adhesion molecule-1 (ICAM-1) and E-selectin mediate leukocyte-endothelial interaction central to atherogenesis; a meta-analysis confirmed that CPAP therapy significantly reduces both in OSA patients (Tian et al., 2021; Fiedorczuk et al., 2023). Endothelin-1 (ET-1), the most potent endogenous vasoconstrictor, is elevated in OSA in proportion to hypoxic severity, and CPAP attenuates this (Kosacka and Brzecka, 2021; Lin et al., 2019).

Proprotein convertase subtilisin/kexin type 9 (PCSK9), synthesized principally in the liver and kidney, degrades hepatic LDL receptors, elevating circulating LDL-cholesterol, and independently upregulates ICAM-1 and VCAM-1 on endothelial cells (Horton et al., 2009; Punch et al., 2022). PCSK9 may represent the most promising early biomarker of screening-detected hypertension (Charkiewicz et al., 2022). The relationship between PCSK9 and IH remains unexplored. OSA and IH are also increasingly recognized as drivers of renal injury, with ROS-mediated tubular damage and intrarenal Renin-angiotensin-aldosterone system (RAAS) activation producing interstitial inflammation and tubular vacuolisation in experimental models (Abuyassin et al., 2018; Mahajan and Gowrishankar, 2024).

Vitamin D, a pleiotropic secosteroid hormone, exerts cardiovascular, immune, and renal regulatory effects through genomic and non-genomic pathways via the widely distributed vitamin D receptor (VDR) (Zmijewski, 2022). Vitamin D deficiency is linked with increased OSA severity and adverse CV outcomes (Loh and Sukor, 2023; Schiza et al., 2024). Mechanistically, vitamin D upregulates eNOS (Kim et al., 2020), suppresses RAAS to attenuate ET-1 secretion and ICAM-1 expression (Wimalawansa, 2018), inhibits NF-κB-driven adhesion molecule production (Bozic et al., 2015), and protects renal tubular epithelium through anti-apoptotic and anti-fibrotic pathways (Gembillo et al., 2020). An emerging inverse association between 25(OH)D and PCSK9 suggests possible VDR-mediated PCSK9 transcriptional suppression (Punch et al., 2022), yet no controlled experimental study has examined this in an IH context.

Despite these mechanistic insights, no published study has systematically examined the dose-dependent capacity of vitamin D_3_ supplementation to modulate a full panel of circulating endothelial biomarkers ICAM-1, E-selectin, ET-1, NO, and PCSK9 alongside renal histopathological outcomes in a controlled OSA model. Drawing on our previous experimental study (Alotaibi et al., 2026), we hypothesized that vitamin D deficiency amplifies IH-induced endothelial activation and renal injury, and that dose-dependent cholecalciferol supplementation attenuates these changes proportionally to dose and hypoxia severity.

## 2 Methods

### 2.1 Study design and ethical approval

This study is a secondary analysis of a controlled experiment conducted at the Prince Naif bin Abdulaziz Health Research Center, King Saud University (KSU), Riyadh, Saudi Arabia (May–July 2025). All protocols were approved by the KSU Institutional Animal Care and Use Committee (IRB: KSU-SE-24-20) and adhered to international animal welfare standards (ARAC, 2025). Full details of the animal model, IH chamber, and supplementation procedures are reported in our previous study (Alotaibi et al., 2026); the present account summarizes only what is necessary to contextualize the biochemical and histopathological outcomes.

### 2.2 Animals and experimental groups

Fifty-six adult male Sprague-Dawley rats (8–10 weeks; 180–250 g) were randomly allocated to seven groups of eight after 1 week of acclimatization: (1) normoxic control (CTRLg); (2) vitamin D-deficient (VDDg); (3) severe IH + vitamin D deficiency (SIH-VDDg); (4a) severe IH + high-dose VD3 (SIH-VDHDg); (4b) severe IH + low-dose VD3 (SIH-VDLDg); (5a) moderate IH + high-dose VD3 (MIH-VDHDg); (5b) moderate IH + low-dose VD3 (MIH-VDLDg).

### 2.3 Vitamin D deficiency induction and supplementation

Deficiency was induced over 3 weeks using a vitamin D-free AIN-93G diet (Dyets Inc., USA; #119266) with restricted UV-B exposure, confirmed by serum 25(OH)D < 25 ng/mL. Oral cholecalciferol (2800 IU/mL; Medical Union Pharmaceuticals, Egypt) was then administered by gavage for 2 weeks at 37.5 μg/kg/day (high-dose) or 25 μg/kg/day (low-dose). These high- and low-dose regimens were selected based on the study by Hodgkinson et al. (2018), which showed that oral vitamin D_3_ within this range produces dose-stratified increases in serum 25(OH)D in growing male Sprague–Dawley rats without inducing toxicity over a comparable supplementation period. Daily oral gavage was used to ensure consistent vitamin D_3_ exposure independent of dietary intake (Hodgkinson et al., 2018). Serum 25(OH)D was also measured at study endpoint using the same ELISA platform (MBS3808712, MyBioSource).

### 2.4 Intermittent hypoxia protocol

IH was delivered in a custom acrylic chamber (150 cm × 60 cm × 100 cm) controlled by a Raspberry Pi 4 microcontroller (Roshan et al., 2023). Severe IH: FiO_2_ (fraction of inspired oxygen) 5%–8% for 60 s, normoxia (21% oxygen concentration) for 120 s, 15–20 cycles per hour. Moderate IH: FiO_2_ 8%–14% for 90 s, normoxia 90 s, ∼20 cycles/h. Exposures lasted 8 h/day for 14 consecutive days, simulating OSA nocturnal pathophysiology.

### 2.5 Blood collection and serum preparation

Blood was collected by retro-orbital sinus technique after 12-h fasting under sevoflurane anesthesia (4% induction, 3% maintenance; IACUC, 2023). Serum was prepared by centrifugation at 3,000 rpm for 18 min at 4 °C (Sigma 3–30 K centrifuge; Sigma, 2025) and stored at −80 °C. All assays were run in duplicate; hemolyzed or lipaemic samples were excluded (Adamiec-Mroczek et al., 2025).

### 2.6 Serum biomarker quantification

Five endothelial and vascular biomarkers were quantified by rat-specific ELISA under expert supervision at KSU College of Pharmacy. Absorbance was read on a Synergy HTX Multi-Mode Microplate Reader (Agilent BioTek, VT, USA). All assays were run in duplicate. Kit details are summarized in Table 1.

**TABLE 1:** ELISA kits are used for serum biomarker quantification.

Biomarker	Unit	Kit/Manufacturer	Catalogue No.	Detection wavelength
ICAM-1	pg/mL	Abcam (Waltham, MA, USA)	ab100763	450 nm
E-Selectin (sE-Sel)	pg/mL	Abcam (Waltham, MA, USA)	ab100763	450 nm
Endothelin-1 (ET-1)	pg/mL	Abcam (Waltham, MA, USA)	ab133030	450 nm
Nitric Oxide (NO)	µmol/L	Abcam (Waltham, MA, USA)	ab65328	540 nm (Griess)
PCSK9	ng/mL	MyBioSource (San Diego, CA, USA)	MBS2515956	450 nm
25-Hydroxyvitamin D [25(OH)D]	ng/mL	MyBioSource (San Diego, CA, USA)	MBS3808712	450 nm

All assays employed the sandwich ELISA principle except NO, which used a two-step colorimetric Griess reaction. Serum 25(OH)D was measured using a separate kit (MBS3808712, MyBioSource).

### 2.7 Renal histopathological assessment

After euthanasia (sevoflurane overdose, 5%–7%; IACUC, 2023), kidneys were bilaterally excised, formalin-fixed (10% neutral-buffered, ≥24 h), paraffin-embedded, and sectioned at 5 µm (Leica rotary microtome). H&E staining followed standard deparaffinization, hematoxylin (4 min), and eosin (2 min) protocols using automated stainers (Dunn et al., 2024; Rieger et al., 2021). Slides were examined under a BX50 microscope (Olympus, Japan) by a pathologist fully blinded to group allocation.

Semi-quantitative histopathological scoring assessed three parameters interstitial inflammatory cell (IIC) infiltration (0 none, 1 minimal, 2 mild), glomerular morphology (0 preserved, 1 altered), and tubular epithelial status (0 normal, 1 vacuolised) using ordinal criteria adapted from Chow et al. (2024), Mahajan and Gowrishankar (2024), and Wen et al. (2020). Scoring was performed by manual visual assessment, by a board-certified pathologist examining each slide under a BX50 microscope (Olympus, Japan).

### 2.8 Statistical analysis

Data was analyzed in IBM SPSS Statistics, Version 28.0 (IBM, 2025). Normality of each biomarker within groups was assessed using the Shapiro–Wilk test, and homogeneity of variances was evaluated using Levene’s test (Ranganathan, 2021). Multivariate homogeneity of covariance matrices was examined by Box’s M test, and Mahalanobis distances were inspected to identify and exclude multivariate outliers prior to multivariate analysis (Ranganathan, 2021). Between-group differences in the combined endothelial biomarker profile (NO, ICAM-1, PCSK9, ET-1, E-selectin) were then assessed using one-way multivariate analysis of variance (MANOVA). When the overall MANOVA was significant, we performed univariate ANOVA for each biomarker followed by least significant difference (LSD) *post hoc* tests for pairwise comparisons. Effect size was reported as partial eta squared (η^2^_p_), in line with recommended practice for reporting intervention magnitude (Sullivan and Feinn, 2012). Renal histopathological scores were summarized descriptively. p < 0.05 was considered statistically significant.

## 3 Results

### 3.1 Multivariate analysis of endothelial biomarkers

One-way MANOVA demonstrated a highly significant overall group effect upon the combined biomarker profile (Table 2). All four test statistics yielded p < 0.001, with partial η^2^ ranging from 0.653 to 0.782, confirming that vitamin D status, dose, and hypoxia severity jointly modulated circulating endothelial markers.

**TABLE 2:** One-way MANOVA results (df = 6, 49).

Test statistic	Value	F	Hypothesis df	Error df	p	Partial η^2^
Pillai’s trace	3.920	7.54	66	264	<0.001	0.653
Wilks’ lambda	0.142	7.98	66	260	<0.001	0.671
Hotelling’s trace	4.801	8.43	66	248	<0.001	0.695
Roy’s largest root	3.377	13.11	12	44	<0.001	0.782

p = significance level; Partial η^2^ = partial eta squared.

### 3.2 Univariate biomarker effects

All five biomarkers showed significant group effects at p < 0.001 (Table 3), with uniformly large effect sizes (η^2^ = 0.694–0.958). Nitric oxide exhibited the highest sensitivity (η^2^ = 0.958), followed by ICAM-1 (0.927) and PCSK9 (0.921).

**TABLE 3:** Univariate ANOVA results for each serum biomarker (df = 6, 49), ranked by effect size.

Biomarker	Unit	F (df = 6,49)	p	Partial η^2^	Group effect direction
Nitric oxide (NO)	µmol/L	187.06	<0.001	0.958	Lowest in VDD + IH; restored by HD
ICAM-1	ng/mL	104.28	<0.001	0.927	Highest in VDD + IH; reduced by HD
PCSK9	ng/mL	95.03	<0.001	0.921	Peak in VDD + IH; near-control with HD-Sev
ET-1	pg/mL	29.12	<0.001	0.781	Elevated in VDD + IH; dose-dependent ↓
E-selectin	ng/mL	18.49	<0.001	0.694	Elevated in VDD + IH; HD-Sev ≈ control

HD, High-dose vitamin D_3_ (37.5 μg/kg/day); VDD + IH, vitamin D deficiency + intermittent hypoxia; Sev, Severe IH.

### 3.3 Descriptive statistics of serum biomarkers

Table 4 presents descriptive statistics for all five biomarkers across experimental groups. The SIH-VDDg demonstrated the most adverse endothelial profile across every analyte, whilst MIH-VDHDg consistently achieved the closest approximation to control values.

**TABLE 4:** Descriptive statistics (mean ± SD) of serum endothelial biomarkers and serum vitamin D_3._

Group	NO (µmol/L) M ± SD	ICAM-1 (ng/mL) M ± SD	PCSK9 (ng/mL) M ± SD	ET-1 (pg/mL) M ± SD	E-Sel (ng/mL) M ± SD	Vitamin D_3_ (ng/mL) M ± SD
CTRLg	29.36 ± 1.08	111.13 ± 2.23	157.13 ± 3.04	2.84 ± 0.31	1.82 ± 0.12	31.06 ± 5.63
VDDg	18.51 ± 0.57	161.50 ± 2.45	192.38 ± 3.34	3.75 ± 0.58	3.31 ± 0.62	6.95 ± 1.72
SIH-VDDg	14.88 ± 2.16	201.50 ± 21.55	213.50 ± 14.71	5.31 ± 0.81	4.92 ± 0.90	2.94 ± 1.21
SIH-VDHDg	21.10 ± 0.36	150.50 ± 4.57	162.50 ± 2.88	3.60 ± 0.33	2.51 ± 0.49	14.44 ± 5.07
SIH-VDLDg	20.45 ± 0.76	181.00 ± 3.63	173.50 ± 3.59	3.78 ± 0.23	2.80 ± 0.97	17.03 ± 2.00
MIH-VDHDg	23.98 ± 0.90	160.75 ± 3.73	180.75 ± 2.32	4.15 ± 0.24	3.06 ± 0.21	14.45 ± 2.35
MIH-VDLDg	14.60 ± 0.63	200.75 ± 3.58	207.75 ± 3.58	4.98 ± 0.26	3.30 ± 0.59	7.20 ± 1.88

Values are mean ± SD (n = 8 per group) HD, High-dose (37.5 μg/kg/day); LD, Low-dose (25 μg/kg/day). CTRLg, Control; VDDg, Vitamin D deficiency; SIH, Severe IH; MIH, Moderate IH; VDHDg, High-dose supplemented; VDLDg, Low-dose supplemented.

Figure 1 and Supplementary Figure S1 present the biomarker concentrations and normalized profiles across groups, respectively. Supplementary Figure S2 provides a percentage-deviation heatmap, Figure 2 illustrates the inverse NO–ICAM-1 correlation, and Figure 3 shows the dose × severity interaction for each analyte.

**FIGURE 1:**
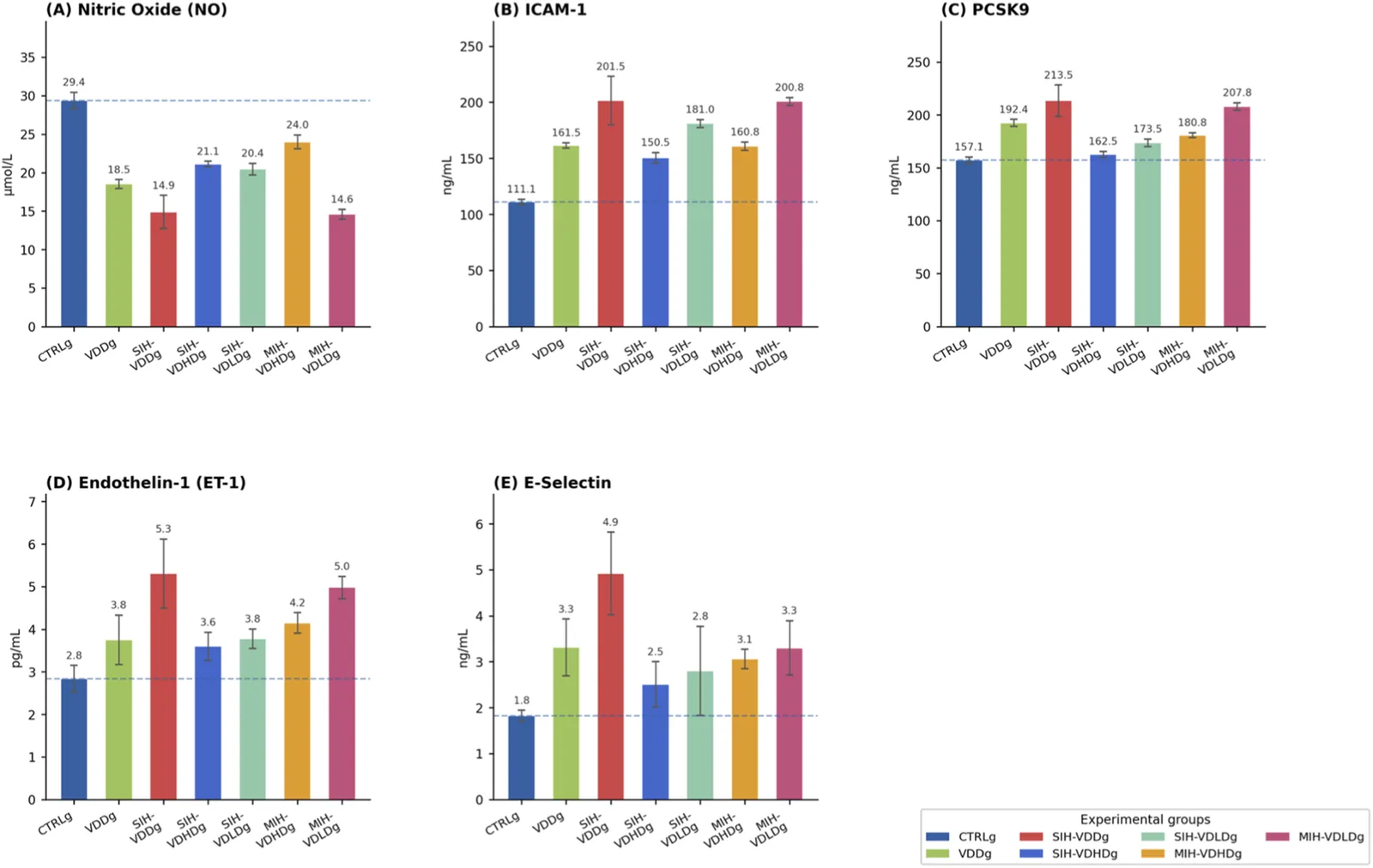
Serum concentrations are presented as mean ± SD (n = 8 per group). **(A)** description of nitric oxide (NO; µmol/L), **(B)** description of ICAM-1 (ng/mL), **(C)** description of PCSK9 (ng/mL), **(D)** description of endothelin-1 (ET-1; pg/mL), and **(E)** description of E-selectin (ng/mL) across all seven experimental groups. Error bars = 1 SD. Dashed reference line = CTRLg mean. All between-group differences p < 0.001. HD = high-dose (37.5 μg/kg/day); LD = low-dose (25 μg/kg/day).

**FIGURE 2:**
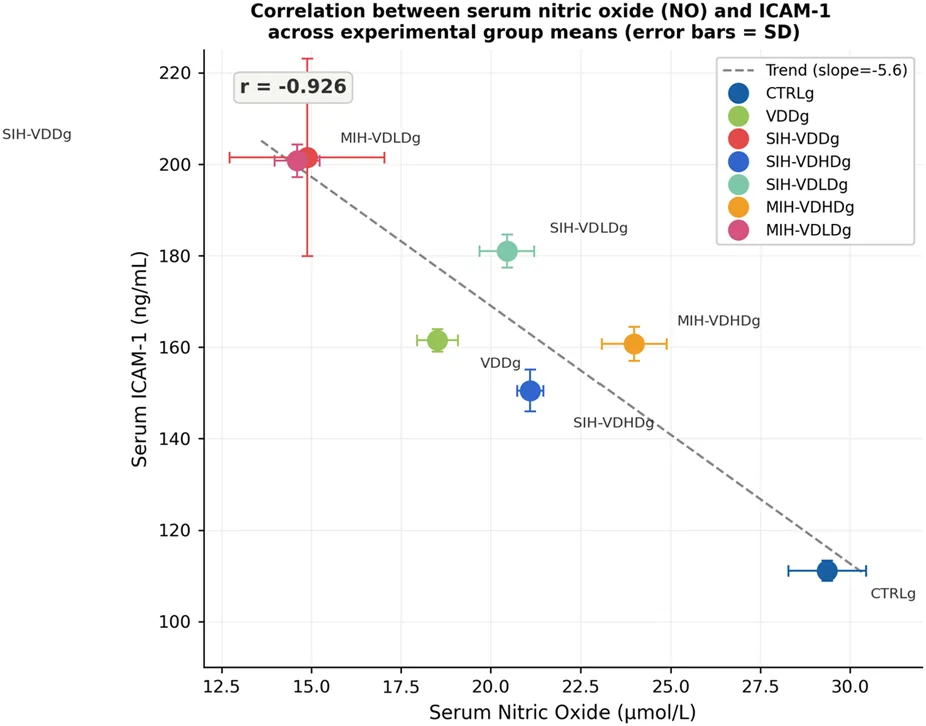
Scatter plot of group-mean NO (µmol/L) vs. ICAM-1 (ng/mL). Error bars = 1 SD. Dashed line = regression. Strong negative correlation (Pearson r annotated) supports a shared eNOS-mediated endothelial mechanism. Color coding is consistent with Figure 1.

**FIGURE 3:**
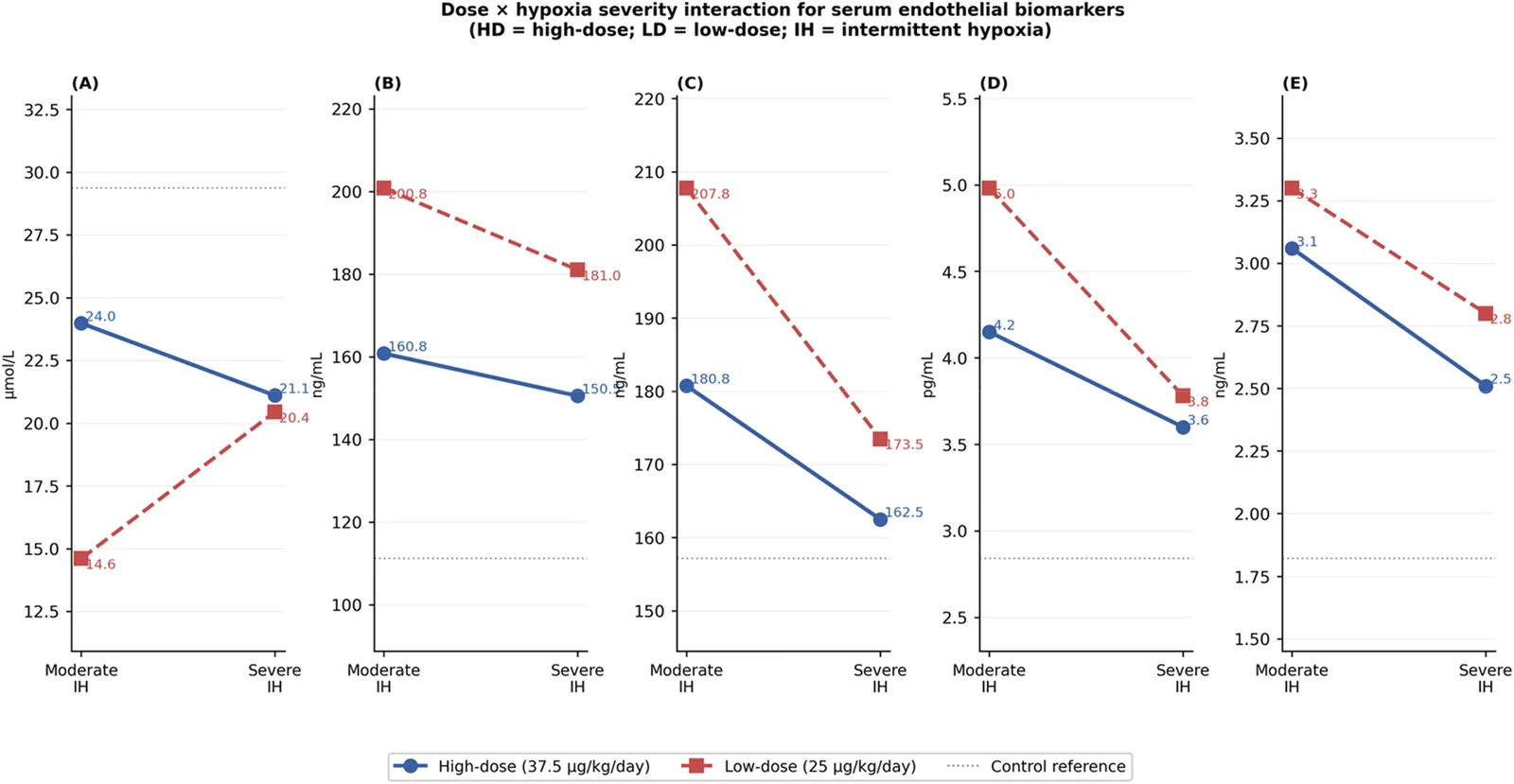
Dose × hypoxia severity interaction for each biomarker. Blue circles (solid): high-dose (37.5 μg/kg/day); red squares (dashed): low-dose (25 μg/kg/day). Dotted horizontal line = CTRLg reference. Divergence between lines under severe IH demonstrates the dose-by-severity interaction. Panels: **(A)** NO; **(B)** ICAM-1; **(C)** PCSK9; **(D)** ET-1; **(E)** E-selectin.

#### 3.3.1 Individual biomarker findings

Nitric oxide demonstrated the highest effect size (η^2^ = 0.958). The CTRLg exhibited M = 29.36 μmol/L (SD = 1.08), declining 36.9% in VDDg (M = 18.51 μmol/L) and 49.3% in SIH-VDDg (M = 14.88 μmol/L). High-dose supplementation under moderate IH (MIH-VDHDg) achieved near-complete restoration (M = 23.98 μmol/L; p > 0.05 vs. CTRLg), whilst low-dose under severe IH (SIH-VDLDg) provided negligible recovery (M = 14.60 μmol/L).

ICAM-1 increased 81.3% above control in SIH-VDDg (M = 201.50 ng/mL, SD = 21.55). High-dose supplementation substantially reduced ICAM-1 (SIH-VDHDg: 150.50 ng/mL; MIH-VDHDg: 160.75 ng/mL), although neither fully normalised to control (p < 0.001). Low-dose under moderate IH (MIH-VDLDg: 200.75 ng/mL) was statistically indistinguishable from untreated SIH-VDDg (p > 0.05).

PCSK9 rose 35.9% above control in SIH-VDDg (M = 213.50 ng/mL). Notably, high-dose supplementation under severe IH (SIH-VDHDg) reduced PCSK9 to 162.50 ng/mL statistically comparable to CTRLg (p > 0.05; mean difference = +5.37 ng/mL), representing the most pharmacologically complete normalisation observed in the study.

ET-1 peaked at 5.31 pg/mL in SIH-VDDg (87.0% above control). High-dose under severe IH (SIH-VDHDg) reduced ET-1 to 3.60 pg/mL, comparable to VDDg alone (p > 0.05). MIH-VDLDg (4.98 pg/mL) approached SIH-VDDg levels (p > 0.05).

E-selectin reached 4.92 ng/mL in SIH-VDDg (170.3% above control). SIH-VDHDg reduced it to 2.51 ng/mL, the only supplementation group not significantly different from CTRLg (p > 0.05; mean difference = +0.69 ng/mL).

### 3.4 Post-hoc pairwise comparisons

Table 5 summarises the key LSD post hoc pairwise contrasts for each biomarker across groups.

**TABLE 5:** Summary of key LSD *post hoc* pairwise contrasts.

Marker	Direction	Key contrast	Mean Δ	p	Interpretation
NO	↑ with VD	VDD + IH < CTRLg; MIH-VDHDg ≈ CTRLg	−14.48 μmol/L (nadir); +9.10 recovery	<0.001	Near-complete restoration with HD-Moderate
ICAM-1	↓ with VD	VDD + IH > all; MIH-VDLDg ≈ VDD + IH	+90 ng/mL above ctrl at peak	<0.001	Incomplete normalisation even at HD
PCSK9	↓ with VD	SIH-VDHDg ≈ CTRLg; MIH-VDLDg ≈ VDD + IH	+56 ng/mL at peak; HD-Sev ≈ ctrl	<0.001	Most complete normalisation at HD-Severe
ET-1	↓ with VD	VDD + IH > all; SIH-VDHDg ≈ VDDg	+2.47 pg/mL at peak	<0.001	HD reduces ET-1 to deficiency-only level
E-Sel	↓ with VD	VDD + IH > all; SIH-VDHDg ≈ CTRLg	+3.10 ng/mL at peak	<0.001	Best normalisation with HD-Severe

HD, High-dose (37.5 μg/kg/day); LD, Low-dose; Sev = Severe IH; Mod = Moderate IH; VD, Vitamin D_3_.

### 3.5 Renal histopathological assessment

#### 3.5.1 Semi-quantitative renal scores

**TABLE 6:** Semi-quantitative renal histopathological scores.

Experimental group	IIC score (0–2)	Glomeruli (0–1)	Tubular epithelium (0–1)
CTRLg Control	0 (None)	0 (Preserved)	0 (Normal)
VDDg Vitamin D deficiency	1 (Minimal)	0 (Preserved)	0 (Normal)
SIH-VDDg VDD + Severe IH	2 (Mild)	0 (Preserved)	1 (Vacuolised) *
SIH-VDHDg Severe IH + HD	2 (Mild)	0 (Preserved)	0 (Normal)
SIH-VDLDg Severe IH + LD	2 (Mild)	0 (Preserved)	0 (Normal)
MIH-VDHDg Moderate IH + HD	2 (Mild)	0 (Preserved)	0 (Normal)
MIH-VDLDg Moderate IH + LD	2 (Mild)	0 (Preserved)	0 (Normal)

Semi-quantitative scoring was performed on renal sections from all 8 animals per group, and Table 6 summarizes group-level scores derived from the complete 56-animal dataset. IIC = Interstitial inflammatory cells. HD = 37.5 μg/kg/day; LD = 25 μg/kg/day. * Proximal tubular cytoplasmic vacuolisation present exclusively in SIH-VDDg.

#### 3.5.2 Group-wise histological findings

Figure 4 presents representative H&E-stained kidney sections from three key groups. The CTRLg (Figure 4A) showed normal architecture: preserved glomeruli, no interstitial infiltrate, and homogeneous tubular epithelium. The SIH-VDDg (Figure 4B) demonstrated mild interstitial lymphocytic infiltration around peritubular capillaries and, uniquely among all groups, subtle cytoplasmic vacuolisation of proximal tubular epithelium (arrows), indicating early tubular stress injury. Glomerular architecture remained preserved. The SIH-VDHDg (Figure 4C) showed mild interstitial infiltration comparable to SIH-VDDg, but with complete absence of tubular vacuolisation, confirming that high-dose vitamin D_3_ supplementation protects proximal tubular morphology despite sustained severe IH exposure.

**FIGURE 4:**
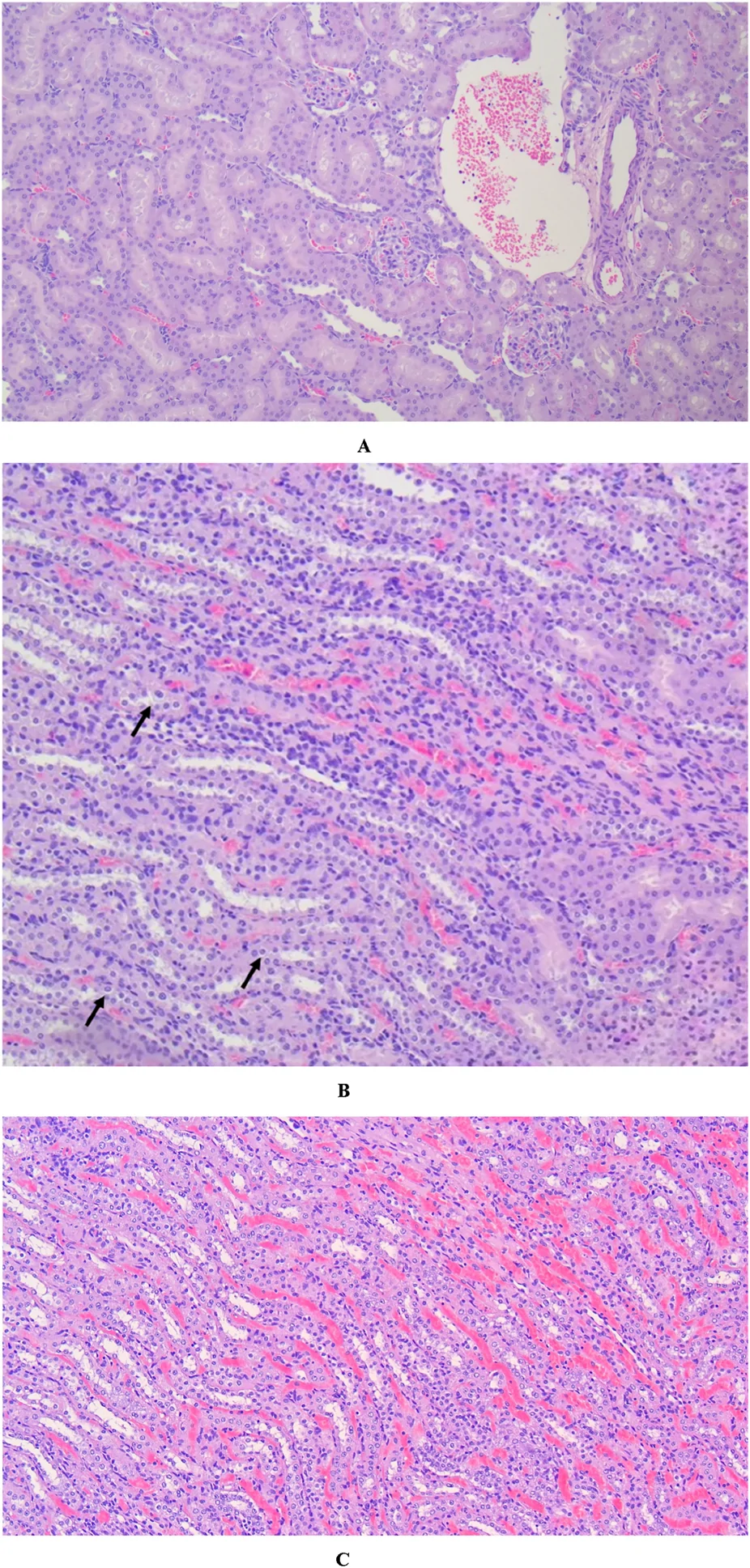
**(A)** Kidney tissue Control group (CTRLg). Normal renal parenchyma with preserved glomerular architecture, no interstitial inflammatory infiltrate, and intact tubular epithelium. Hematoxylin and Eosin, ×100; scale bar = 100 µm. **(B)** Kidney tissue Vitamin D deficiency with severe intermittent hypoxia (SIH-VDDg). Mild interstitial lymphocytic infiltrate around peritubular capillaries and interlobular vessels. Subtle cytoplasmic vacuolisation of proximal tubular epithelium (arrows), indicating early tubular injury. Glomeruli remain preserved. Hematoxylin and Eosin, ×100; scale bar = 100 µm. **(C)** Kidney tissue Severe intermittent hypoxia with high-dose vitamin D_3_ supplementation (SIH-VDHDg). Mild interstitial lymphocytic infiltrate persists, but proximal tubular epithelium is entirely normal with no vacuolisation, demonstrating vitamin D_3_-mediated protection of tubular morphology. Glomeruli remain preserved. Hematoxylin and Eosin, ×100; scale bar = 100 µm.

Across all four supplementation groups (SIH-VDHDg, SIH-VDLDg, MIH-VDHDg, MIH-VDLDg), tubular vacuolisation was entirely absent irrespective of dose or hypoxia severity, indicating that even the lower supplementation dose (25 μg/kg/day) was sufficient to prevent tubular vacuolisation. No significant morphological distinction was detectable between high-dose and low-dose subgroups. Glomerular architecture remained preserved in all seven groups.

### 3.6 Integrative biomarker–histology correlation

The SIH-VDDg exhibiting peak ICAM-1 (201.50 ng/mL), ET-1 (5.31 pg/mL), PCSK9 (213.50 ng/mL), E-selectin (4.92 ng/mL), and trough NO (14.88 μmol/L) was the sole group to demonstrate proximal tubular vacuolisation. This concordance supports a mechanistic link between systemic endothelial inflammatory activation and renal tubular oxidative stress mediated through reduced NO bioavailability and elevated ET-1-driven intrarenal vasoconstriction. Conversely, high-dose supplementation groups demonstrating the greatest biochemical recovery also showed complete preservation of tubular morphology, reflecting the multi-target renoprotective action of vitamin D_3_.

## 4 Discussion

This study demonstrates that vitamin D deficiency combined with intermittent hypoxia produces a coherent pattern of endothelial dysfunction and renal tubular injury, and that dose-dependent vitamin D_3_ supplementation attenuates these changes through coordinated effects on nitric oxide bioavailability, adhesion molecule expression, ET-1 signaling, and PCSK9 modulation. Among the five biomarkers, NO demonstrated the highest effect size (η^2^ = 0.958), reflecting its central role as the primary endothelial vasoprotective molecule. The 49.3% NO depletion in SIH-VDDg results from two converging mechanisms: vitamin D deficiency impairs VDR-mediated eNOS transcription and PI3K/Akt-dependent phosphorylation (Kim et al., 2020; Ni et al., 2014), whilst IH-generated ROS scavenge bioavailable NO and uncouple eNOS (Lavie, 2015; Mahmoud et al., 2019). High-dose supplementation under moderate IH restored NO to near-control levels (p > 0.05), consistent with vitamin D_3_-mediated eNOS re-coupling and antioxidant pathway restoration (Andrukhova et al., 2014). The strong negative correlation between group-mean NO and ICAM-1 (Figure 2) corroborates the mechanistic link between NO suppression and NF-κB-driven adhesion molecule upregulation (Tran et al., 2022).

Both adhesion molecules showed group-dependent elevations consistent with the literature: vitamin D receptor deletion directly upregulates ICAM-1 and VCAM-1 (Bozic et al., 2015), and CPAP meta-analyses confirm that reducing IH lowers circulating ICAM-1 and E-selectin (Tian et al., 2021; Pak et al., 2014). The differential recovery pattern of E-selectin normalized fully at high-dose severe IH (p > 0.05 vs. control), whilst ICAM-1 remained significantly elevated even at best, reflecting distinctions in their regulatory mechanisms: E-selectin expression is acutely and reversibly cytokine-driven, whereas ICAM-1 is governed by more sustained NF-κB, AP-1, and Sp1 transcriptional regulation, likely requiring longer supplementation for complete normalization (Ilinčić et al., 2017). In metabolically heterogeneous human populations, Mirzavandi et al. (2020) found no effect of vitamin D on E-selectin in type 2 diabetes, suggesting that systemic metabolic disease may limit endothelial responsiveness that is more readily observable in our uniform rat model.

ET-1 peaked at 87% above control in SIH-VDDg, consistent with dual upregulatory mechanisms under vitamin D deficiency: derepression of RAAS elevates angiotensin II, which stimulates endothelial ET-1 synthesis, whilst loss of direct VDR-mediated suppression of the prepro-ET-1 promoter further amplifies production (Arfian et al., 2018; Kosacka and Brzecka, 2021). High-dose supplementation under severe IH reduced ET-1 to levels comparable to VDDg alone (p > 0.05), confirming that high-dose vitamin D_3_ neutralized the hypoxic contribution to ET-1 excess. The persisting imbalance between NO and ET-1 across all groups provides the molecular substrate for the hemodynamic elevations reported previously (Alotaibi et al., 2026).

The modulation of PCSK9 by vitamin D_3_ is the most pharmacologically novel finding of this study. PCSK9 exceeded control by 35.9% in SIH-VDDg, likely reflecting hepatic and endothelial upregulation through IH-driven NF-κB and IL-6 signaling alongside loss of VDR-mediated PCSK9 promoter suppression (Punch et al., 2022; Meszaros and Bikov, 2022). Three converging mechanisms may underline the observed vitamin D_3_-mediated PCSK9 suppression: direct VDR binding to VDRE sequences in the PCSK9 promoter, reduction of cytokine-driven hepatic PCSK9 expression through NF-κB inhibition, and indirect suppression via RAAS attenuation (Charkiewicz et al., 2022). Notably, PCSK9 normalization was most complete at high-dose severe IH (SIH-VDHDg ≈ CTRLg; p > 0.05), an unexpected severity-specific pattern that may reflect proportionally greater VDR-mediated suppression against the more intense hepatic inflammatory stimulus of severe IH, warranting mechanistic investigation including direct measurement of hepatic LDLR expression in future studies.

The exclusive occurrence of proximal tubular vacuolisation in SIH-VDDg reflects the threshold effect of combined vitamin D deficiency and severe IH upon tubular energetics. Tubular vacuolisation represents early mitochondrial swelling and endoplasmic reticulum stress secondary to ROS-driven mitochondrial permeability transition in proximal tubular cells, the most metabolically demanding tubular segment (Mahajan and Gowrishankar, 2024; Abuyassin et al., 2018). The perivasal lymphocytic infiltration in VDDg without hypoxia is consistent with VDR-loss-mediated NF-κB-driven renal chemokine upregulation, as documented by Chow et al. (2024) and Gonçalves et al. (2014). The complete absence of vacuolisation in all supplemented groups at both doses demonstrates that the renal tubular protective threshold is lower than that required for full biomarker normalization, with important implications for minimum effective dosing in OSA management.

Collectively, the data supports a four-target protective cascade for vitamin D_3_: eNOS upregulation restoring NO bioavailability, which in turn suppresses NF-κB-driven ICAM-1 and E-selectin expression, whilst concurrent RAAS and VDR-mediated signaling attenuates ET-1 production and reduces PCSK9 promoter activity. These mechanisms are mutually reinforcing: restored NO inhibits ICAM-1 transcription, reduced oxidative stress prevents eNOS uncoupling, and lowered RAAS activity simultaneously reduces ET-1 and PCSK9 (Wimalawansa, 2018). The dose-by-severity interaction whereby high-dose supplementation consistently outperformed low-dose, and moderate IH permitted greater recovery than severe IH reflects the pharmacological principle that protective efficacy is contextually determined by the competing oxidative burden imposed by hypoxia.

### 4.1 Limitations

Supplementation lasted only 2 weeks and did not fully restore serum 25(OH)D, likely limiting the magnitude of biomarker normalization observed. Only male rats were studied; sex differences in RAAS activity, vitamin D metabolism, and endothelial responsiveness may affect generalizability. Inflammatory cytokines (TNF-α, IL-1β, IL-6) were not directly measured, limiting upstream mechanistic attribution. The NO–ICAM-1 correlation is based on group means rather than individual values, which constrains individual-level inference. In addition, we did not measure renal hypoxia-signaling or oxidative stress markers such as hypoxia-inducible factor-1α (HIF-1α) or xanthine oxidase activity, nor did we apply immunohistochemical staining for lymphocyte subsets (e.g., CD3, CD45). As a result, the present study cannot definitively distinguish the relative contributions of overt hypoxic–ischemic injury versus predominantly oxidative stress–mediated tubular damage, and future work should incorporate HIF-1α, complementary hypoxia and injury markers, and IHC-based inflammatory cell quantification to refine the mechanistic interpretation of the observed histological changes.

## 5 Conclusion

Vitamin D deficiency combined with intermittent hypoxia in an OSA rat model produces a reproducible, multi-dimensional pattern of endothelial dysfunction, simultaneous NO suppression, adhesion molecule and ET-1 upregulation, and PCSK9 amplification accompanied by renal interstitial inflammation and proximal tubular vacuolisation. High-dose vitamin D_3_ supplementation (37.5 μg/kg/day) significantly attenuates this endothelial and renal injury profile, achieving near-complete normalization of NO and PCSK9, meaningful suppression of ICAM-1, ET-1, and E-selectin, and complete prevention of tubular vacuolisation, in a dose- and hypoxia-severity-dependent manner. Low-dose supplementation (25 μg/kg/day) provides partial biochemical protection but prevents tubular vacuolisation at both doses.

The modulation of PCSK9 by vitamin D_3_ is a pharmacologically novel finding with potential implications for OSA-associated dyslipidemia and vascular inflammation. Together with the hemodynamic findings of our previous study (Alotaibi et al., 2026), these results provide comprehensive multi-system experimental support for vitamin D_3_ supplementation as a cost-effective adjunctive strategy against OSA-related endothelial and renal complications. Future work should prioritize longer supplementation durations, female animal inclusion, upstream cytokine measurement, hepatic PCSK9 pathway characterization, and ultimately, well-designed randomized controlled trials in human OSA populations.

## Data Availability

The raw data supporting the conclusions of this article will be made available by the authors, without undue reservation.

## Supplementary material

SUPPLEMENTARY FIGURE S1
Normalised biomarker profiles across experimental groups.

SUPPLEMENTARY FIGURE S2
Percentage-deviation heatmap of serum biomarkers across groups.

## Glossary

25(OH)D: 25-hydroxyvitamin D
ANOVA: Analysis of variance
ARAC: Animal Research Advisory Committee
CPAP: Continuous positive airway pressure
CTRLg: Control group
CV: Cardiovascular
eNOS: Endothelial nitric oxide synthase
ELISA: Enzyme-linked immunosorbent assay
ET-1: Endothelin-1
FiO2: Fraction of inspired oxygen
H&E: Hematoxylin and eosin
HD: High-dose (37.5 μg/kg/day)
IACUC: Institutional Animal Care and Use Committee
ICAM-1: Intercellular adhesion molecule-1
IH: Intermittent hypoxia
IRB: Institutional Review Board
KSU: King Saud University
LDL: Low-density lipoprotein
LDLR: Low-density lipoprotein receptor
LD: Low-dose (25 μg/kg/day)
LSD: Least significant difference
MANOVA: Multivariate analysis of variance
MIH: Moderate intermittent hypoxia
MIH-VDHDg: Moderate IH + high-dose vitamin D3 group
MIH-VDLDg: Moderate IH + low-dose vitamin D3 group
NF-κB: Nuclear factor kappa B
NO: Nitric oxide
OSA: Obstructive sleep apnea
PCSK9: Proprotein convertase subtilisin/kexin type 9
PNHRC: Prince Naif bin Abdulaziz Health Research Center
RAAS: Renin-angiotensin-aldosterone system
ROS: Reactive oxygen species
SD: Standard deviation
sE-Sel: Soluble E-selectin
SIH: Severe intermittent hypoxia
SIH-VDDg: Severe IH + vitamin D deficiency group
SIH-VDHDg: Severe IH + high-dose vitamin D3 group
SIH-VDLDg: Severe IH + low-dose vitamin D3 group
VD: Vitamin D
VDD: Vitamin D deficiency
VDDg: Vitamin D deficiency group
VDR: Vitamin D receptor
VCAM-1: Vascular cell adhesion molecule-1
VDHDg: Vitamin D high-dose supplemented group
VDLDg: Vitamin D low-dose supplemented group
