# Supplementary material for: Dose-dependent vitamin D3 supplementation attenuates endothelial dysfunction and renal injury in an obstructive sleep apnea rat model
Source: Front Pharmacol. 2026 Sep 15;17:1847011. doi: 10.3389/fphar.2026.1847011 (PMC13619501; doi:10.3389/fphar.2026.1847011)
Supplement: Supplementary file 1 [file Supplementaryfile1.docx]

**Supplementary Figure S1.** *Radar plot of endothelial biomarker profiles normalized to control group mean (100%; dashed circle). The NO axis is inverted (outward = worsening). Five groups plotted: CTRLg (blue), SIH-VDDg (red; maximal derangement), SIH-VDHDg (amber), MIH-VDHDg (dark green; closest to control), and MIH-VDLDg (pink).*

**Supplementary Figure S2.** *Heatmap of percentage deviation from control for each biomarker across all groups. Red = worsening; blue = improvement. NO axis sign-inverted for directional consistency. The SIH-VDDg row shows uniformly deep red coloring, confirming maximal endothelial dysfunction.*
